# Lst4, the yeast Fnip1/2 orthologue, is a DENN-family protein

**DOI:** 10.1098/rsob.150174

**Published:** 2015-12-02

**Authors:** Angela Pacitto, David B. Ascher, Louise H. Wong, Beata K. Blaszczyk, Ravi K. Nookala, Nianshu Zhang, Svetlana Dokudovskaya, Tim P. Levine, Tom L. Blundell

**Affiliations:** 1 Department of Biochemistry, University of Cambridge, 80 Tennis Court Road, Cambridge CB2 1GA, UK; 2 Department of Cell Biology, UCL Institute of Ophthalmology, 11-43 Bath Street, London EC1V 9EL, UK; 3 CNRS UMR 8126, Université Paris-Sud 11, Institut Gustave Roussy, 114, rue Edouard Vaillant, Villejuif 94805, France

**Keywords:** Lst4, DENN-family, BHD syndrome, Lst7, Fnip1/2, folliculin

## Abstract

The folliculin/Fnip complex has been demonstrated to play a crucial role in the mechanisms underlying Birt–Hogg–Dubé (BHD) syndrome, a rare inherited cancer syndrome. Lst4 has been previously proposed to be the Fnip1/2 orthologue in yeast and therefore a member of the DENN family. In order to confirm this, we solved the crystal structure of the N-terminal region of Lst4 from *Kluyveromyces lactis* and show it contains a longin domain, the first domain of the full DENN module. Furthermore, we demonstrate that Lst4 through its DENN domain interacts with Lst7, the yeast folliculin orthologue. Like its human counterpart, the Lst7/Lst4 complex relocates to the vacuolar membrane in response to nutrient starvation, most notably in carbon starvation. Finally, we express and purify the recombinant Lst7/Lst4 complex and show that it exists as a 1 : 1 heterodimer in solution. This work confirms the membership of Lst4 and the Fnip proteins in the DENN family, and provides a basis for using the Lst7/Lst4 complex to understand the molecular function of folliculin and its role in the pathogenesis of BHD syndrome.

## 1. Introduction

Birt–Hogg–Dubé (BHD) syndrome is a rare autosomal-dominantly inherited disorder which predisposes patients to benign tumours of the hair follicle (fibrofolliculomas), lung cysts that give rise to pneumothoraces and kidney tumours [1]. This disorder arises from germline mutations in the *FLCN* gene [2], and much effort over the past decade has gone into unravelling the molecular function of its protein product, folliculin (Flcn). Flcn has been shown to be involved in numerous signalling pathways, including the mechanistic target of rapamycin complex 1 (mTORC1) pathway [3–5], energy sensing through AMP-activated protein kinase (AMPK) [3,6], the transforming growth factor *β* pathway [7], autophagy regulation [8,9] and Wnt signalling [10] among others, though a precise understanding of its role at the molecular level remains to be achieved. Flcn is also known to have two paralogous binding partners, the Flcn interacting partners Fnip1 and Fnip2, which interact independently with Flcn [3,11,12]. This Flcn/Fnip (either Flcn/Fnip1 or Flcn/Fnip2) complex has been recently reported to be involved in amino acid sensing through regulation of the Rag GTPases at the lysosomal membrane and therefore controlling signalling through mTORC1 [13,14].

We previously determined the structure of the C-terminal domain of Flcn [15], which provided the first insights into the potential molecular function of the protein. This structure revealed that Flcn is homologous to the differentially expressed in normal and neoplastic (DENN) tissue family of proteins, with which it shares low sequence similarity. The core DENN family proteins are known to be GTP-exchange factors (GEFs) for the Rab family of GTPases [16], with the members of each DENN subfamily regulating a single Rab GTPase at a different cellular location. Based on the known association between Rabs and DENN family proteins, we previously showed that the Flcn C-terminal domain has *in vitro* GEF activity, in particular towards Rab35 however, while this domain does possess GEF activity, Rab35 may not be its *in vivo* target [15]. More recently, conflicting reports have emerged about the mode of GTPase interaction of the Flcn/Fnip complex, with both GEF and GTPase activating protein (GAP) activity proposed towards Rag A/B and Rag C/D, respectively [13,14].

**Figure 1.**
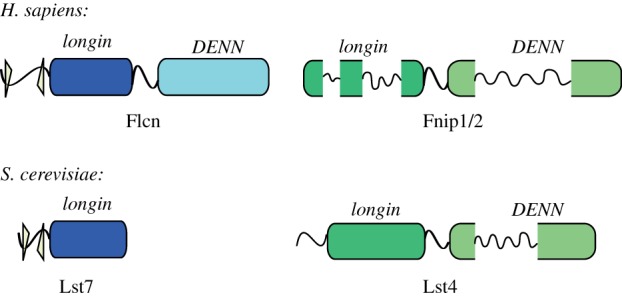
Predicted architecture of the human Flcn and Fnip1/2 proteins and the yeast Lst7 and Lst4 proteins. The yellow triangles indicate the putative zinc-binding cysteine and histidine residues conserved in both Flcn and Lst7. Both Fnip1/2 and Lst4 are predicted to have large unstructured insertions within the globular longin and DENN domains.

The complete DENN module found in the DENN family of proteins comprises an N-terminal longin domain, commonly found in a variety of trafficking proteins [17], and a C-terminal DENN domain. Bioinformatics analysis of DENN revealed that the family is larger than previously thought. While the core members are reasonably well conserved at the sequence level, the wider family is much more divergent and could only be detected by a more sensitive fold-recognition approach [18,19]. These studies proposed that Fnip1 and Fnip2 were also divergent DENN family proteins, albeit with large unstructured insertions within the globular longin and DENN domains (figure 1).

The conservation of Flcn throughout the eukaryotic lineage implies an important functional role of this protein. Interestingly, *Saccharomyces cerevisiae* has a shorter form of Flcn, Lst7, which is homologous with the putative zinc-finger and longin domain of the Flcn N-terminal region (figure 1). The *LST7* gene was originally identified in a screen for genes that are synthetic lethal with a temperature-sensitive allele of *SEC13* [20]. Sec13 is a multitasking protein being part of the nuclear pore complex [21], the COPII vesicle coat [22] and the Seh1-associated complex [23]. It was shown that Lst7 is involved in trafficking of the general amino acid permease (Gap1p) to the cell surface, when cells are grown on a poor nitrogen source. Yeast lacking Lst7 can grow on certain toxic amino acid analogues owing to the absence of Gap1p at the cell surface [20].

With the motivation to understand more about the Flcn/Fnip complex, we sought to investigate their putative yeast orthologues Lst7 and Lst4. We solved the structure of the Lst4 longin domain, thereby confirming it as a bona fide member of the DENN family. Additionally, we show that Lst4 interacts with Lst7, not through its longin domain but through its DENN domain, and we demonstrate that the Lst7/Lst4 complex exists as a 1 : 1 heterodimer. In an analogous fashion to the Flcn/Fnip complex, Lst7/Lst4 localizes to the vacuolar membrane, which is particularly pronounced when the cells are starved of carbon and to a lesser extent nitrogen. This work provides a further piece in the puzzle towards understanding the molecular architecture of the Flcn/Fnip complex and provides a foundation for studying this complicated disease-associated protein complex in yeast.

## 2. Results and discussion

### 2.1. Structure of the Lst4 longin domain

**Figure 2.**
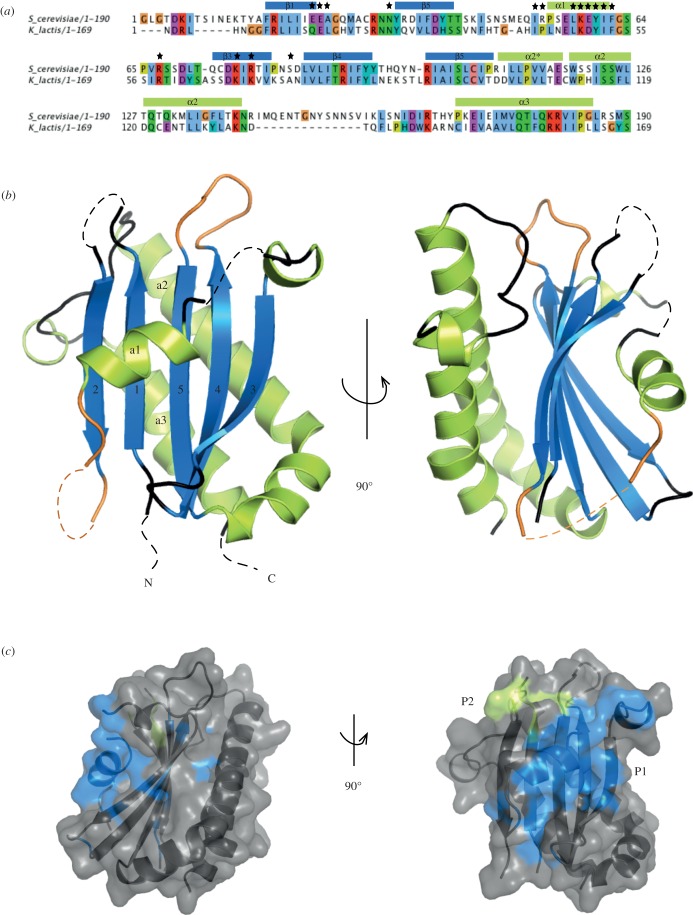
X-ray crystal structure of the longin domain from *K. lactis* Lst4. (*a*) Sequence alignment of *S. cerevisiae* and *K. lactis* Lst4 longin domain sequences, coloured in Clustal colours with secondary structure elements annotated. Black stars indicate conserved surface residues. (*b*) The crystal structure of the *K. lactis* Lst4 longin domain has the classical longin domain fold. Dashed lines indicate the loop regions that were not visible in the electron density and loops coloured orange indicate the equivalent regions in fnip1/2, which have large unstructured insertions. (*c*) Consurf analysis of the Lst4 longin domain structure identified two conserved surface patches P1 and P2, which are conserved in other longin domains.

In order to confirm that Lst4 and therefore Fnip1/2 belong to the DENN family, we decided to pursue structure determination of this protein. We chose the N-terminal region of the putative yeast orthologue Lst4 for crystallization as this proposed longin domain is not disrupted by long insertions that are predicted to be unstructured, as is the DENN domain and the Fnip1/2 longin domain (figure 1). Initial crystals of the Lst4 longin domain (aa 103–292) from *S. cerevisiae* were obtained however, although they diffracted well, these crystals were not reproducible. Analysis with Phenix.xtriage of the native data indicated that they were perfectly twinned, and derivative preparation proved unfeasible. In order to overcome this, the Lst4 longin domain from *Kluyveromyces lactis,* a closely related budding yeast, was expressed, purified and crystallized. The domains share 33% sequence identity (figure 2*a*) and as can be seen from the alignment, the *K. lactis* longin domain is more compact than that found in *S. cerevisiae*.

The *K. lactis* longin domain (aa 58–226) crystallized in the P4_2_2_1_2 space group with four molecules in the asymmetric unit (AU). Phases for structure solution were obtained from a SAD experiment on a single gold derivative crystal prepared by soaking the native crystals overnight in 1 mM KAu(CN)_2_. The final structure was determined at 2.14 Å resolution by molecular replacement of a higher resolution native dataset. Several loops were missing in the electron density, between *β*1–*β*2, *β*2–*α*1 and *α*1–*β*3, in addition to nine residues at the N-terminus and 13 residues at the C-terminus, most likely owing to their inherent flexibility. Superposition of the four molecules within the AU using BATON [24] showed that there was very little difference between the chains, with the RMSD across the C*α* backbone relative to chain D for chains A, B and C being 0.646, 0.883 and 0.883 Å, respectively. For analysis/figure purposes, chain D was chosen as a representative, given the similarity of all four chains in the AU.

The structure reveals the classical architecture of a longin domain, which is the first domain of the complete DENN module (figure 2*b*). The domain is made up of a core five-strand β-sheet, with one short *α*-helix (*α*1) traversing the face of the concave side and the longer *α*2 and *α*3 packed against the convex side. As mentioned previously, both Fnip1 and Fnip2 have two additional large unstructured insertions within this domain [19] (figure 2*b*, highlighted in orange). These lie between *β*2 and *α*1 and between *β*4 and *β*5, with lengths of 61 and 162 amino acids, respectively. The function of these regions is at present unknown; however, given the large number of serine/threonine residues in these insertions, it may be that they are involved in the regulation of the complex by phosphorylation.

The packing interfaces within the Lst4 longin crystals include a local dyad axis between the chains C and B, and D and A (electronic supplementary material, figure S1*a*). The residues that are in this crystal-packing interface mainly lie on *β*3 and form hydrophobic interactions. This may be explained by the fact that it is this region that interfaces with the DENN domain in the DENN1B structure [25], and in a structure of the whole protein this region would probably be buried in the interface with the C-terminal DENN domain. The four molecules seen in the crystal structure do not represent a biologically relevant assembly, as an analytical ultracentrifugation experiment indicated that the protein was monomeric in solution (electronic supplementary material, figure S1*b*).

Interestingly, a DALI search [26] of the Lst4 longin domain gave as the top hit human synbindin (Protein Data Bank (PDB) ID: 2zmv), which has an insertion within the longin domain in the same position as the first insertion in Fnip1/2 (*β*2–*α*1). This insertion in synbindin folds into an atypical PDZ domain [27], which indicates it is not unprecedented for the longin domains to be split with other functional regions inserted in them. There are at present, however, no experimental longin domain structures that contain insertions within the second Fnip1/2 insertion region, *β*4–*β*5. As secondary structure predications indicate that these regions are likely to be disordered, their analysis by X-ray crystallography seems unlikely.

Comparison of this longin domain with other members of the longin family reveals some interesting features. It would be expected that this longin domain would be most similar to that of the DENN1B, given that this is the only structure of a longin domain from a DENN protein that has been determined. While the domains superimpose reasonably well with a RMSD across the C*α* backbone of 2.99 Å (electronic supplementary material, figure S2), there are various extensions in loop regions relative to one another. An entirely novel feature, which is not present in any other previously determined longin domain structure, is the kinked helical extension to *α*2. A recently published longin domain structure of the yeast TRAPP III associated protein Tca17 [28] has an extension in this region like Lst4, however it does not form such a well-defined helix.

ConSurf [29] analysis of the structure identified two conserved surface regions of the Lst4 longin domain, which were mapped onto a surface representation of the Lst4 longin structure (figure 2*c*). The first patch (P1) mainly comprises residues from *α*1 and several that lie on the loop that precedes *β*3. This conservation is seen in other longin domains and has previously been called the ‘A region’, which is responsible for intra- and inter-molecular interactions in a variety of longin domain proteins [17]. The second smaller patch, P2, comprises residues in the *β*1–*β*2 loop.

### 2.2. Lst7 interacts with the DENN domain of Lst4

**Figure 3.**
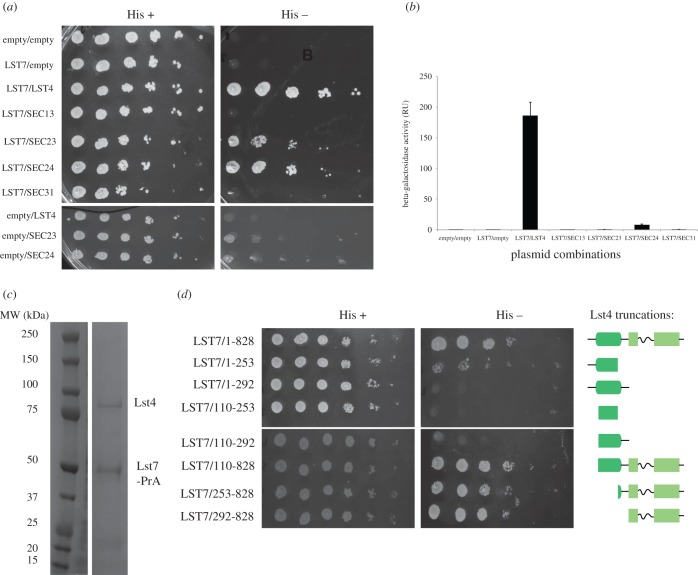
Lst7 interacts with the putative yeast Fnip orthologue Lst4. (*a*) Yeast two-hybrid experiments demonstrate the interaction of Lst4 with Lst7. The COPII proteins were used as a positive control and Lst7 shows a weak interaction with Sec23/Sec24. (*b*) A beta-galactosidase assay corroborates the yeast two-hybrid results from figure 1*a*. (*c*) Genomically tagged Lst7-protein A co-immunoprecipitates with Lst4, all bands identified by mass spectrometry. (*d*) Yeast two-hybrid experiments confirm that the DENN domain of Lst4 is able to interact with Lst7 and that the longin domain of Lst4 is not part of the interacting region.

We sought to demonstrate the interaction of Lst4 with Lst7, to validate its designation as a true orthologue of the Flcn/Fnip complex and to investigate the regions responsible for interaction. This was approached using a yeast two-hybrid methodology. In the absence of a positive control, we also tested the interaction of Lst7 with the COPII vesicle coat proteins, as Lst7 was shown in a high-throughput study to interact with Sec24 [30]. Lst7 interacts strongly with Lst4 and also displays a weak interaction with Sec23 and Sec24 (figure 3*a*). Beta-galactosidase assays of the transformed strains further corroborated these findings (figure 3*b*). It has previously been reported that it is the C-terminal domain (DENN domain) of Flcn that interacts with Fnip1 [3]. As there is no C-terminal DENN domain in Lst7 (figure 1), our demonstration that Lst7 exists in a complex with the putative yeast Fnip orthologue Lst4 suggests that the interaction between Flcn and Fnip1/2 may not involve the Flcn DENN domain.

We confirmed the yeast two-hybrid results by generating C-terminal protein A (Pr-A) genomically tagged strains of *LST7* and *LST4* and performing immunoprecipitations with IgG-coated magnetic beads, in buffers of varying stringency. Consistently, MALDI mass spectrometry analysis confirmed that Lst7-PrA co-immunoprecipitated with Lst4 (figure 3*c*). The interaction was observed even under stringent conditions such as 0.5 M NaCl (electronic supplementary material, figure 3*a*), suggesting a strong interaction between the two proteins. In the reciprocal immunoprecipitation, Lst4-PrA co-immunoprecipitated with Lst7, although the band was not as distinctly visible as that of Lst4 in the Lst7 immunoprecipitation high peptide counts and SPECTRUM Mi11 score for Lst7 were obtained (electronic supplementary material, figure 3*b*).

In order to narrow down the region of Lst4 that interacts with Lst7, we made several constructs of truncated Lst4 based on the putative domain boundaries (figure 1) and tested for interaction using the yeast two-hybrid method. We hypothesized that this interaction would be mediated through the dimerization of the longin domains found in both Lst7 and Lst4 [19,31]. However, we observed that it is the DENN domain region of Lst4 that mediates the interaction with Lst7 (figure 3*d*). This is corroborated by Baba *et al.* [3], who showed that residues 300–1166 of Fnip1 are required for binding to Flcn. This truncation comprises the full DENN domain (residues 498–1066) of Fnip1 [19] and was the only truncation of Fnip1 tested that showed any interaction with Flcn.

### 2.3. Lst4 and Lst7 localize to the vacuole in response to nutrient starvation

**Figure 4.**
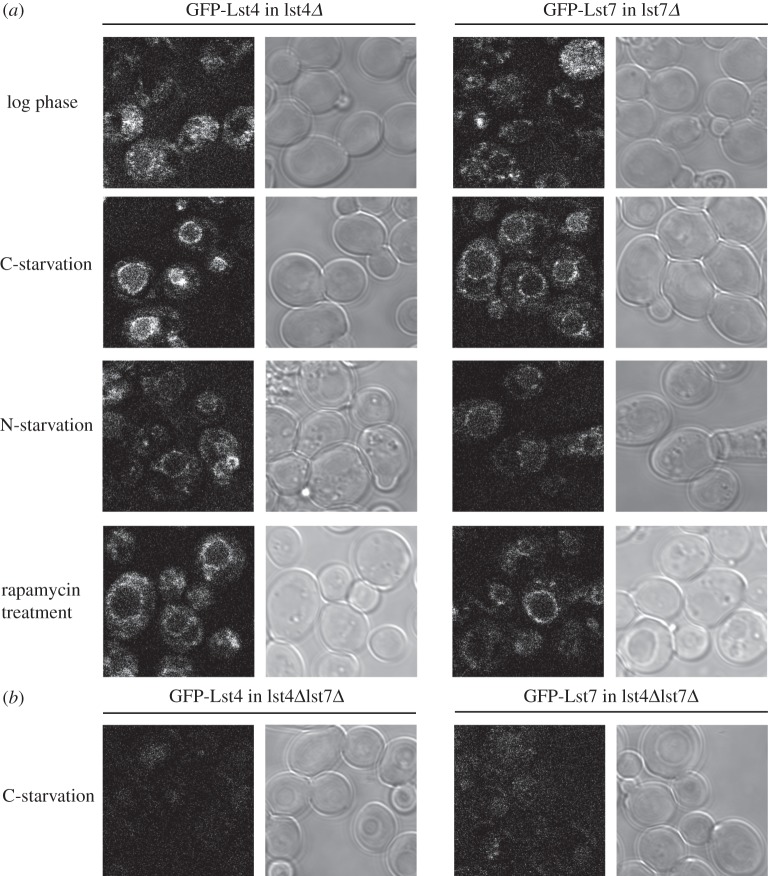
GFP-Lst4 and GFP-Lst7 localize to the vacuolar membrane under conditions of nutrient stress. (*a*) In log phase, GFP-Lst4 and GFP-Lst7 are localized diffusely in the cytoplasm with some weak vacuolar targeting. Upon a variety of nutrient stresses such as carbon starvation, nitrogen starvation or rapamycin treatment both proteins localize to the vacuolar limiting membrane in a punctate fashion. (*b*) In the absence of each other, in an lst4Δlst7Δ background, GFP-Lst4 and GFP-Lst7 fail to localize to the vacuolar membrane when the cells are carbon starved. In general, the GFP fluorescence is reduced, which may suggest the individual proteins are not stable in the absence of each other.

To investigate the cellular location of Lst4 and Lst7, plasmids containing GFP-LST4 and GFP-LST7 under the control of their endogenous promoters were transformed into the BY4741 haploid strains lacking the endogenous protein (i.e. lst4Δ and lst7Δ). The GFP-tagged proteins were localized diffusely throughout the cytoplasm when the cells were in log phase, with a small proportion of cells showing weak vacuolar targeting. The vacuolar targeting became much clearer upon starvation of the cells for glucose (figure 4*a*). It was observed that this localization was not uniform but, instead, there was punctate staining around the vacuolar limiting membrane. In addition, other nutrient stresses, such as nitrogen starvation and rapamycin treatment, also increased vacuolar targeting but not as strongly as carbon starvation. Both GFP-Lst4 and GFP-Lst7 showed similar localization in response to each stimulus as would be expected from proteins that exist as a complex. Interestingly, when either GFP-Lst4 or GFP-Lst7 was expressed in the absence of the other, no GFP signal above background or distinct localization pattern was observed, even when the cells were carbon starved (figure 4*b*). This suggests that in the absence of each other the individual proteins are no longer stable and are degraded, consistent with them forming an obligate complex. Encouragingly, these experiments replicate in yeast what has been seen in a mammalian system with Flcn/Fnip localizing to the lysosome, although the predominant signal tested for the latter has been amino acid starvation [13,14]. These localization experiments indicate that in response to nutrient stress there is regulated recruitment of the Lst7/4 complex to the vacuolar membrane, a site where many Lst4 and Lst7 potential downstream protein interaction partners (EGO complex, TORC1) also localize.

### 2.4. Lst7 and Lst4 form a 1 : 1 heterodimer

**Figure 5.**
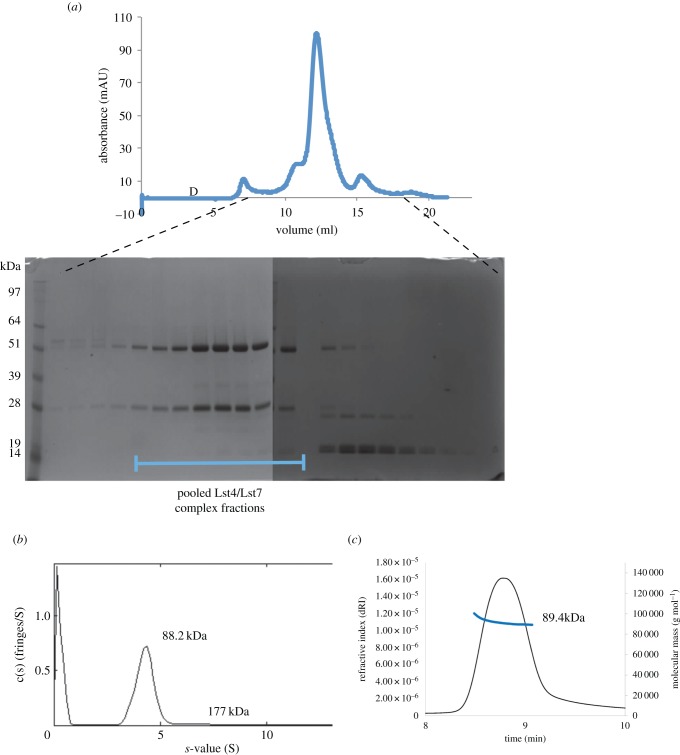
The engineered Lst7/Lst4 complex forms a 1 : 1 heterodimer. (*a*) Purification of the Lst7/Lst4 complex. Superdex 200 elution profile of Lst7/Lst4 and SDS–PAGE gels of the elution fractions. (*b*,*c*) Analytical ultracentrifugation and SEC-MALS experiments show that the molecular weight of the *K. lactis* Lst7/Lst4 complex is equivalent to that predicted for a 1 : 1 heterodimer (89.3 kDa).

The yeast two-hybrid experiments, immunoprecipitations and localization studies indicated that Lst4 and Lst7 displayed a strong interaction that is likely to be constitutive. To further investigate the complex, as with our crystal structure, we again used the *K. lactis* orthologues, as they proved to be more stable than their *S. cerevisiae* counterparts. We co-expressed Lst4 and Lst7 constructs in *Escherichia coli*. In order to facilitate expression we engineered the DENN domain of Lst4 to remove the 200 residue unstructured insertion, and we were able to purify the complex (figure 5*a*). This engineered Lst4 was still able to interact with Lst7, showing that this region has not evolved to mediate the interaction with Lst4, but must have other functions. One possibility is that the long insertion within the DENN domain may play a role in regulating the function of the Lst7/Lst4 complex. The large number of serine residues within this region suggests that modification by phosphorylation may be possible and this is something we have previously observed upon overexpression of the native complex in *S. cerevisiae* (2013, unpublished data).

The recombinant complex was investigated by size-exclusion multiangle X-ray scattering and analytical ultracentrifugation, and this demonstrated that the Lst7/Lst4 complex exists as a 1 : 1 heterodimer comprising two longin domains and one DENN domain (figure 5*b*,*c*).

In considering the implications of the longin domain structure in the context of the heterodimeric Lst7/Lst4 complex, we looked to the Denn1B : Rab35 structure where the longin domain is involved only in minimal contacts at the interface with the GTPase [25] with the majority of interface residues lying within the C-terminal DENN domain. It is the *β*3–*β*4 loop and several residues that lie on *α*1 of the longin domain of Denn1B that are within 5 Å of Rab35, and this overlaps with the previously described ‘A region’. It has been observed that this binding region differs from that observed in other longin–GTPase protein complexes [25]. In other cases of longin domain : GTPase interactions, it was proposed that the mechanism of activity relied on the *β*1–*β*2 loop, which in Lst4 seems to show some degree of conservation (P2 in figure 2*c*) [17,32]. This raises an intriguing possibility; given that in the Lst7/Lst4 complex we have shown that the Lst7 longin domain interacts with the Lst4 DENN domain, it is possible that both proteins are required to make a ‘complete’ DENN module. That would leave the Lst4 longin domain available to interact with other as yet undiscovered binding partners. It has been previously hypothesized that in the case of Rab GTPases [33], cascades of signalling can occur in which one Rab can bind the cognate GEF of the downstream Rab, and this leads to the recruitment of the downstream Rab and the signalling cascade proceeds. This scenario could be envisaged for the Lst4/Lst7 complex, in which the longin domain of Lst4 could bind an upstream GTPase, whereas it is the complete DENN module made up of the domains from both proteins, which interact with another GTPase, that could be the target of activity. Another possibility is that the longin domain of Lst4 could be involved in interacting with Snf1, the yeast AMPK orthologue, as Fnip1 has been reported to bind AMPK [3].

An alternative hypothesis regarding GTPase interaction modes is possible, which is that the binding of Lst7 to the Lst4 DENN domain occludes the putative GTPase binding surface regions (if Lst4 was superimposed on the Denn1B : Rab35 structure). It has previously been proposed that the Flcn/Fnip complex is able to act as a GAP towards Rag C/D [14]. This runs counter to the notion that DENN family proteins are GTPase exchange factors. However, if the binding of Lst7 to Lst4 somehow prevents it from acting as a conventional DENN family protein, then the idea that the complex is in fact a GAP may be more likely.

### 2.5. Concluding remarks

Previously reported bioinformatics predictions had shown that Lst7 is the yeast Flcn orthologue, given the evident sequence homology, and had suggested that there might be a sequence relationship between Lst4 and Fnip1/2 [31]. These studies also predicted that Fnip1/2 and therefore Lst4 were members of the DENN family. In order to confirm this, we solved the structure of the N-terminal region of Lst4 and showed it contains a longin domain. To confirm the orthologous relationship of Lst7/Lst4 with Flcn/Fnip we demonstrated that Lst4 interacts with Lst7 through its putative DENN domain, thus providing experimental evidence supporting the role of Lst4 as the Fnip1/2 orthologue. The Flcn/Fnip complex has been shown previously to relocate to the lysosome [13,14,34] in response to amino acid starvation. Similarly, we have shown that Lst7 and Lst4 are targeted to the vacuole when the cells are starved of carbon, and to a lesser extent nitrogen. Therefore, this replicates and expands upon what has previously been seen in a mammalian system.

While this manuscript was under review, Péli-Gulli *et al.* [35] published a study of the Lst7/Lst4 complex, which demonstrated similar findings regarding the vacuolar localization of the complex in response to amino acid starvation and rapamycin treatment. Their data confirmed the existence of the Lst7/Lst4 complex, which is orthologous to the Flcn/Fnip complex, and also demonstrated that in order to see the vacuolar relocalization upon nutrient stress the partner protein had to be present, further reinforcing our hypothesis that Lst7/Lst4 form an obligate complex. They also showed that the complex has GAP activity towards Gtr2, as has been previously proposed for the Flcn/Fnip2 complex towards Rag C/D, the Gtr2 human orthologues [14]. It will require further research to understand the mechanisms behind this activity, given that DENN family proteins are commonly found to possess GEF activity. A crystal structure of the intact Lst7/Lst4 complex and the possibility of seeing how these domains are arranged spatially may inform whether the DENN fold of Lst4 can act as a GEF in the same way as Denn1B or could explain the molecular basis of the GAP activity.

**Figure 6.**
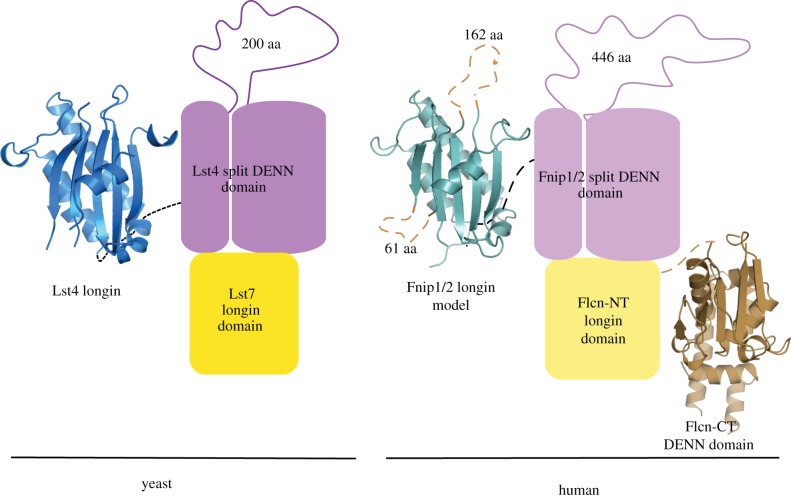
Proposed model of the organization of the Lst7/Lst4 complex and by homology the Flcn/Fnip complex. Lst7 interacts with the split DENN domain of Lst4, therefore we propose that the Flcn-N terminal (NT) longin domain is involved in the interaction with the split-DENN domain of Fnip1/2. The longin domain of Fnip1/2 has been modelled on the Lst4 longin domain structure determined in this study (PDB ID: 4ZY8). The dashed orange lines indicate the unstructured insertions present within the Fnip1/2 longin domain. The Flcn-C-terminal (CT) DENN domain was determined previously (PDB ID: 3V42), and this domain is absent in the yeast orthologue Lst7.

Finding a targeted treatment for BHD syndrome rests on understanding the precise molecular function of the Flcn/Fnip complex. The confirmation that Lst4 and therefore Fnip1/2 is a DENN family protein means that the Flcn/Fnip complex is unique in its composition of two DENN family proteins, an arrangement that has not been reported for other members of this protein family. This complex is involved in the cellular response to nutrient status, and we propose a model by which these proteins interact in both yeast and human systems (figure 6). Understanding how the two DENN modules are arranged and how this could impact their function will shed further light on their role in BHD. The work presented here demonstrates that with regards to these proteins the similarity between yeast and humans is closer than previously thought. This opens up the opportunity to study Lst7/Lst4 both structurally and biologically in a lower eukaryote, in order to draw inferences about the Flcn/Fnip complex. This avenue of research could provide the insights required to address some of the questions posed by the aforementioned conflicting studies.

## 3. Material and methods

### 3.1. Lst4 longin domain protein expression, purification and analytical ultracentrifugation

The Lst4 longin (L4L) domain from *K. lactis* (*aa58–226*) was cloned into pHat5 [36] and expressed with a C-terminal His_6_-tag in BL21 (DE3) Star cells (Life Technologies). Cells were grown up at 37°C to an OD_600_ of 0.6, and protein expression was induced at 18°C for 16 h with 0.5 µM IPTG (final concentration). Cells were harvested by centrifugation and resuspended in 20 mM Tris pH 8.0, 500 mM NaCl and 5 mM *β*-mercaptoethanol before lysis using an Emulsiflex cell disruptor (Avestin). Cell lysate was clarified by centrifugation and the supernatant applied to a His-select affinity column (Sigma). The protein was eluted with 20 mM Tris pH 8.0, 500 mM NaCl, 5 mM *β*-mercaptoethanol and 300 mM imidazole, and was dialysed overnight into the lysis buffer. Size-exclusion chromatography was performed on a Superdex 75 16/60 column with a final buffer of 20 mM Tris pH 8.0, 500 mM NaCl, 2.5 mM TCEP. Following this, the protein was concentrated to 12 mg ml^−1^ (*K. lactis*) using VivaSpin 20 10 kDa MWCO centrifugal concentrators (Sartorius) for crystallization.

Analytical ultracentrifugation experiments were performed on a Beckman–Coulter XL-1 ultracentrifuge. Samples were spun at 40 000 rcf at 20°C, and 300 absorbance scans of the protein sample were taken. The data were analysed and fitted using Sedfit [37].

### 3.2. Crystallization of the *K. lactis* Lst4 longin domain

The L4 L domain from *K. lactis* was crystallized in 4 µl hanging drops in a 1 : 1 ratio with 100 mM Bis–Tris, pH 5.8, 3.1 M NaCl, 1.1% butan-1-ol; crystals appeared after 48 h and grew to full size within one week. Crystals were cryoprotected in reservoir solution containing 4 M NaCl, before freezing. Data were collected on native crystals at diamond light source (DLS) beamline I02.

Gold derivative crystals were prepared by soaking the native crystals in reservoir solution containing 1 mM KAu(CN)_2_ overnight. Crystals were backsoaked in reservoir solution for 2 h before cryoprotection and freezing. Derivative data collection was performed at DLS on beamline I03.

### 3.3. Determining the *K. lactis* Lst4 longin domain structure

Phases were obtained for the *K. lactis* L4L structure using a single gold derivative crystal, from which a single wavelength anomalous diffraction (SAD) dataset at a wavelength of 1.04 Å was collected. The resulting diffraction images were autoprocessed with XDS [38] and scaled with aimless (CCP4). [39]. The calculated Matthews coefficient [40] of 2.09 indicated that the crystal solvent content was 41.1% and there were four molecules in the AU. Using the AutoSol wizard of Phenix [41], 15 Au atoms were located and the figure of merit after density modification was 0.41. The resulting map was submitted to the AutoBuild wizard of Phenix, which built a large proportion of the four chains. At this stage chain C was deemed to be the most complete, so this was manually refined in COOT [42], and the single chain was used as a probe for molecular replacement using the higher resolution native data set at 2.14 Å. Molecular replacement was performed using Phaser within Phenix followed by iterative rounds of refinement using Phenix.refine and manual refinement in COOT. After refinement, the values for *R*_work_ and *R*_free_ were 0.2106 and 0.2522, respectively. The final model contains 99% residues in the preferred regions, 1% in the allowed regions and there are no outlier residues. All figure preparation and structural analysis was done using COOT [42] and Pymol (Schrodinger). For crystallographic statistics, see the electronic supplementary material, tables S1 and S2. The structure has been deposited in the PDB with ID: 4ZY8.

### 3.4. Yeast two-hybrid assays

The *LST7* gene was cloned into pACT2 (Clontech), which contains the GAL activation domain. The *LST4* and *COPII* coat genes were cloned into pLexPD (based on pLexM), which contains the LexA DNA binding domain. Both bait and prey plasmids were transformed into the L40 reporter strain, exploiting the Trp and Leu auxotrophy. Transformants were replated onto fresh plates, and then serial dilutions were performed followed by spotting onto plates with and without histidine. To validate the interaction, the second reporter gene of the L40 strain *LacZ* was used in a standard beta-galactosidase assay, following a previously described protocol [43].

### 3.5. Lst4 and Lst7 protein-A immunoprecipitations

Lst4 and Lst7 were genomically tagged with *Staphylococcus aureus* protein A as described previously [44]. Yeast strains were grown in Wickerham media (0.3% Bacto malt extract (BD Biosciences), 0.3% yeast extract, 0.5% peptone (both Formedium) and 1% d-glucose (Sigma)) to early log phase. Cells were harvested, washed twice with water and once with buffer containing 20 mM K-HEPES (pH 7.4), 1.2% polyvinylpyrrolidone, 1 mM DTT, 1 : 200 of protease inhibitor cocktail (PIC solution, Sigma) and harvested by centrifugation. Cell lysis was achieved by cryogenic grinding as previously described [45].

Immunoprecipitations were performed using a protocol developed by Alber *et al.* [45]. The following buffers were used for extraction of protein complexes: electronic supplementary material figure S1*e*—1 M ammonium acetate pH 7.0, 1 mM DTT, 1% Triton X-100, 1 : 100 solution P (0.04% pepstatin A, 2% phenylmethanesulfonyl fluoride in absolute ethanol), 1 : 100 protease inhibitor cocktail (PIC solution, Sigma P8340); electronic supplementary material, figure S3*a*—20 mM K/HEPES pH 7.4, 110 mM potassium acetate, 2 mM MgCl_2_, 1 mM DTT, 0.1% Tween-20, 1% Triton-X100, 75–500 mM NaCl, 1 : 100 solution P, 1 : 100 PIC; electronic supplementary material, figure S3*b*—TC250 (40 mM Tris pH 8.0, 250 mM sodium citrate, 150 mM NaCl, 2 mM EDTA, 0.1% Tween-20, 1% Triton-X100, 1 : 100 solution P, 1 : 100 PIC). Immunoprecipitated proteins were eluted with 0.5 M ammonium hydroxide and 0.5 mM EDTA, and the elution fractions were dried overnight in a Speedvac. The resulting dried pellets were resuspended in loading dye and run on 4–12% Bis–Tris gels (Life Technologies). Protein bands were identified by MALDI-fingerprinting or LC–MS/MS by the PNAC facility of the Department of Biochemistry, University of Cambridge, and the Proteomic Platform of Institut Gustave Roussy (Villejuif, France).

### 3.6. Fluorescence microscopy

GFP-LST4 and GFP-LST7 were cloned into the CEN/*URA3* plasmid pRS416. To ensure expression at endogenous levels, the promoters of both genes were cloned from yeast genomic DNA and inserted upstream of the *LST4* and *LST7* genes. The resulting constructs were individually transformed into haploid BY4741 strains lacking the endogenous *LST4* and *LST7* genes, i.e. lst4Δ and lst7Δ or both genes lst4Δlst7Δ. The transformed strains were grown to mid-log phase in SC—URA media (0.17% YNB with ammonium sulfate + casamino acids + tryptophan + 2% glucose). For glucose starvation, the cells were spun down and after washing twice with C-starvation media (SC–URA–glucose); they were resuspended again in C-starvation media and incubated for 1 h at 30°C. For N-starvation, yeast nitrogen base without amino acids and ammonium sulfate was prepared + 2% glucose, but with addition of amino acids required for auxotrophic growth. As for the N-starved cells, they were washed twice with N-starvation media and were grown for 1 h. For rapamycin treatment, cells were grown up in SC + 2% glucose–URA and treated with rapamycin 200 ng ml^−1^ for 1 h. Live cells were imaged by confocal microscopy (AOBS SP2; Leica) at room temperature (63× NA 1.4 objective) using LCS software (Leica) for acquisition. Images were handled in Photoshop (Adobe), and equivalent adjustments were applied to all images. All yeast strains are listed in the electronic supplementary material, table S3.

### 3.7. Expression, purification and oligomeric state analysis of the Lst7/Lst4 complex

The genes for wt *LST7* and *LST4* from *K. lactis* were synthesized as codon-optimized genes (for *E. coli* expression) by Life Technologies. Full-length wt Lst7 (*K. lactis*) was cloned into pHat5 with a C-terminal Strep tag included in the reverse primer. Lst4 (*K. lactis*) with the boundaries aa58–714 and a deletion of the intra-DENN loop (aa 388–534) was cloned into pOPINS with an N-terminal His_6_-SUMO tag. The two plasmids were expressed in BL21 (DE3) Star cells (Life Technologies) and grown at 37°C until OD_600_ = 0.6–0.8. Cells were cooled to 18°C, and protein expression was induced with 0.5 µM IPTG. The cells were grown overnight, then harvested by centrifugation at 4200 rcf; resuspended in 20 mM Tris pH 7.5, 500 mM NaCl, 5 mM *β*-mercaptoethanol, and lysed by three passes through an Avestin Emulsiflex cell disruptor. The lysate was clarified by centrifugation at 30 000 rcf for 30 min. The resulting supernatant was loaded onto a 4 ml Steptactin (IBA) column pre-equilibrated with lysis buffer, and washed with 20 ml lysis buffer. Bound protein was eluted as 10 × 2 ml fractions with lysis buffer + 2.5 mM d-desthiobiotin. Elution fractions were pooled and loaded onto a 2 ml nickel affinity column (Sigma), and nickel purification was performed as described previously. In order to remove the SUMO affinity tag the nickel elution pool was dialysed overnight into 20 mM Tris, 500 mM NaCl, 5 mM *β*-mercaptoethanol with the addition of Ulp1 protease. The cleaved protein complex was concentrated and injected onto a Superdex 200 13/30 size-exclusion column (GE) pre-equilibrated with 20 mM Tris pH 7.5, 500 mM NaCl, 2.5 mM TCEP. All elution fractions were snap frozen in liquid nitrogen and stored at −80°C. AUC was performed as above, but owing to the lower protein concentration available (0.15 mg ml^−1^), interference data were collected and used for Sedfit analysis of the sedimentation velocity in order to calculate the predicted molecular weight. SEC-MALS analysis was performed to determine the experimental molecular weight of the Lst7/Lst4 complex as previously described [46,47]. The complex (50 µg in 50 µl) was run on a Tosoh TSKgel SuperSW2000 4.6 × 300 mm column equilibrated in PBS at a flow rate of 0.35 ml min^−1^ using a Shimadzu LC-20AD isocratic HPLC coupled to a Dawn Heleos MALS detector and an Optilab T-rEX refractive index detector (Wyatt Technology). The molecular weight was determined using ASTRA 5 software (Wyatt Technologies).

## Supplementary Material

Supplementary Figures and Tables
