# Supplementary material for: Lst4, the yeast Fnip1/2 orthologue, is a DENN-family protein
Source: Open Biol. 2015 Dec 2;5(12):150174. doi: 10.1098/rsob.150174 (PMC4703059; doi:10.1098/rsob.150174)
Supplement: Supplementary Figures and Tables [file rsob150174supp1.docx]

Supplementary Figures


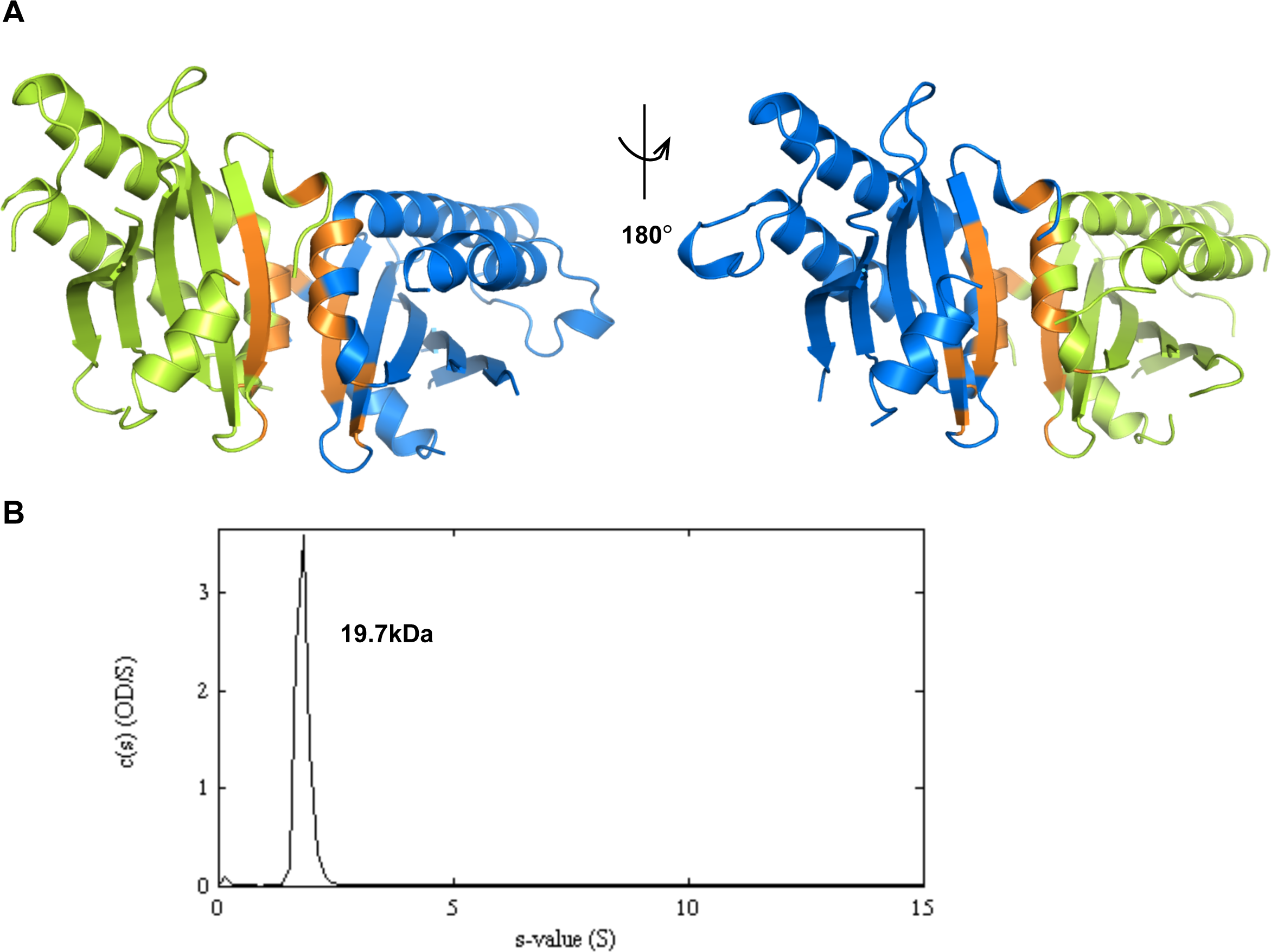


Supplementary Figure 1 Investigation of crystal packing interfaces and oligomeric state of the Lst4 longin domain. A. The crystal-packing interface in the Lst4 longin domain structure mimics the presence of the DENN domain, with the β3 strands of two protomers forming the main interface. B. Analytical ultracentrifugation experiments indicate that the Lst4 longin domain is a monomer in solution and the presence of 4 molecules in the asymmetric unit is a crystal-packing artifact.


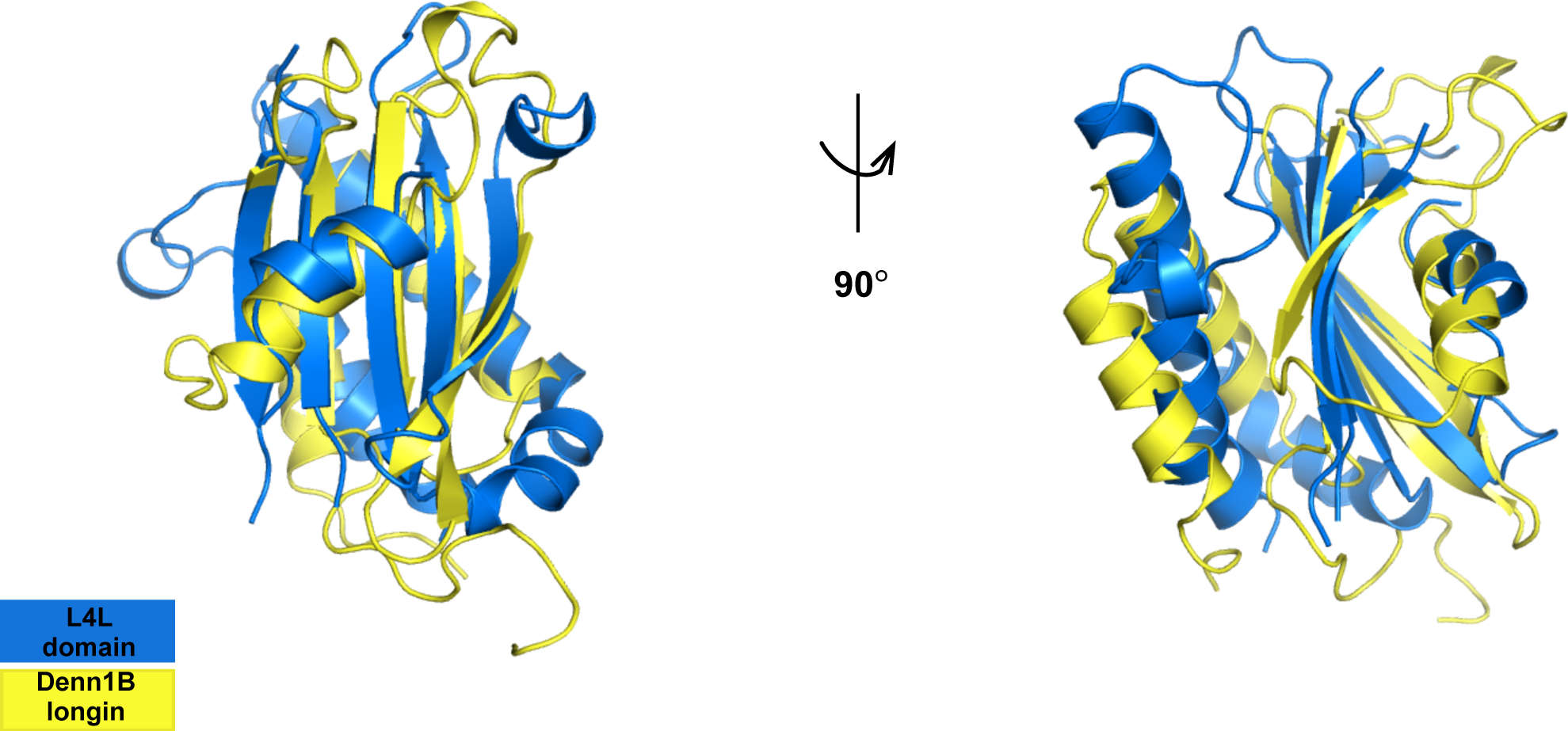


Supplementary Figure 2 Structural superposition of Lst4 longin domain with the longin domain from Denn1B. RMSD over the core, aligned residues is 2.99 Å, and the structures show variability in loop regions, whilst the core β-sheet superimposes well.


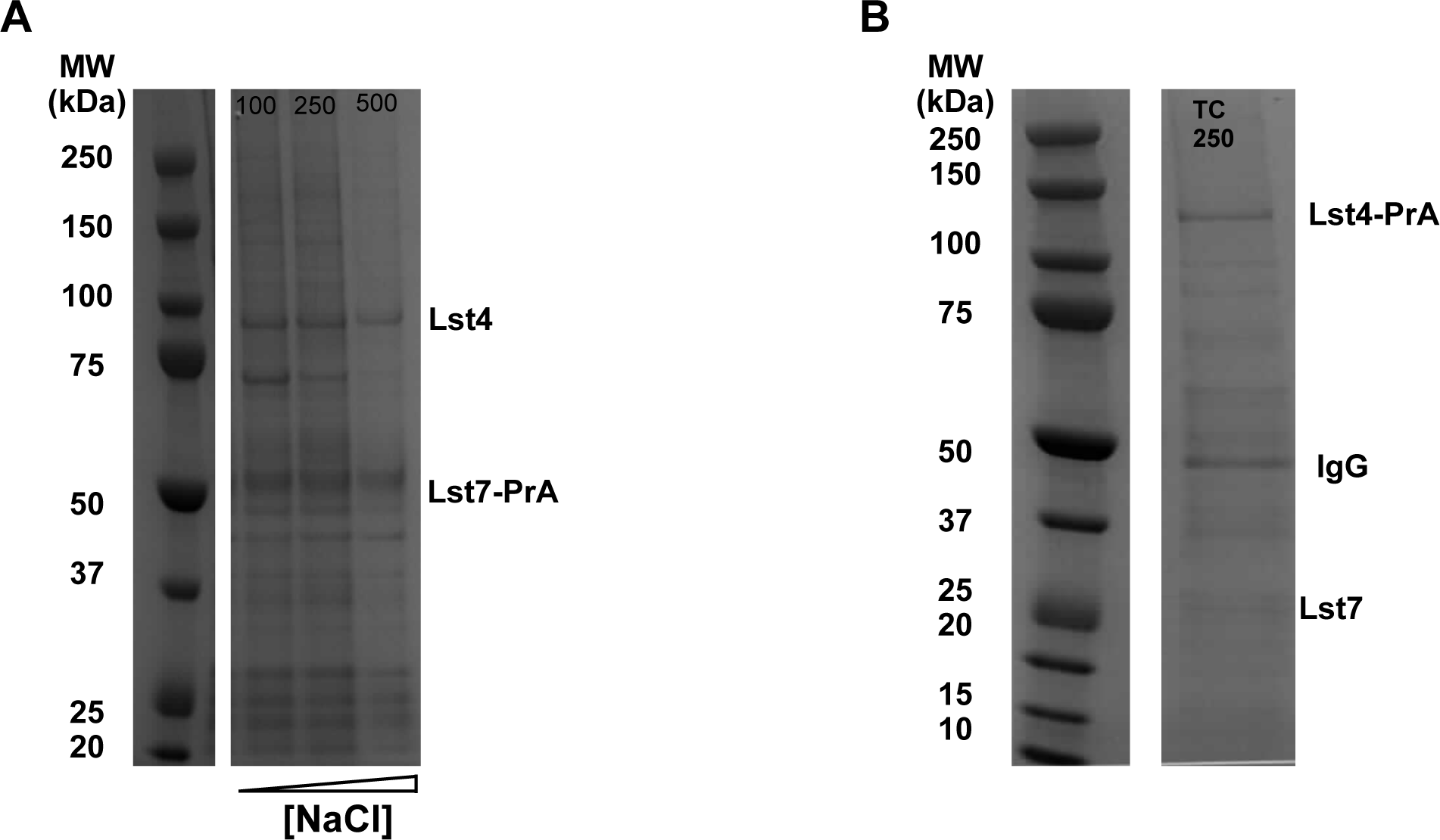


Supplementary Figure 3 Immunoprecipitations of Lst7 and Lst4-Protein A (PrA). A. Lst7-PrA immunoprecipitates Lst4 with increasing NaCl concentration unable to disrupt the interaction (indicated in mM on gel). B. Lst4-PrA co-immunoprecipitates with Lst7. Although not highly visible by Coomassie staining, 9 unique peptides and a Spectrum MiII (d’) score of 147.53 indicate that Lst7 is abundant in the Lst4-PrA immunoprecipitation. (TC250 – buffer used – see methods)

**Supplementary Tables**

**Table S1.** Data collection and phasing statistics.

|  | Lst4 longin native | Lst4 longin Au(CN_2_) derivative |
| --- | --- | --- |
| data collection | | |
| source | diamond Light Source, I02 | diamond Light Source, I03 |
| wavelength (Å) | 0.97949 | 1.03966 |
| space group | P4_2_2_1_2 | P4_2_2_1_2 |
| unit cell parameters | *a* = 120.7 Å, *b* = 120.7 Å, *c* = 93.3 Å, | *a* = 120.2 Å, *b* = 120.2 Å, *c* = 95.6 Å, |
|  | *α* = 90°, *β* = 90°, *γ* = 90° | *α* = 90°, *β* = 90°, *γ* = 90° |
| resolution range (Å) | 35.29–2.14 (2.216–2.14) | 95.61–2.69 (2.76–2.69) |
| observed reflections | 466 857 (65 544) | 288 467 (21 770) |
| unique reflections | 38 603 (5529) | 20 039 (1428) |
| Wilson B (Å^2^) | 39.49 | 55.01 |
| completeness (%) | 100 (100) | 100 (100) |
| multiplicity | 12.1(11.9) | 14.4 (15.2) |
| mean *I*/(*I*) | 20.4 (4.4) | 23.0 (3.5) |
| *R*_meas_ | 0.071 (0.688) | 0.122 (1.000) |
| no. of sites |  | 15 |
| FOM |  | 0.412 |
| overall score |  | 51.97 ± 8.30 |
| density modification | | |
| *R*-factor |  | 0.28 |
| map skew |  | 0.21 |
| correlation of local r.m.s. density |  | 0.84 |

**Table S2.** Refinement statistics.

|  | KLS native |
| --- | --- |
| space group | P4_2_2_1_2 |
| resolution (Å) | 35.29–2.14 |
| no. of reflections (work/test) | (38 550/1858) |
| protein atoms (no. of residues) | 4377 (573) |
| no. of solvent molecules | 174 |
| ions | 0 |
| *R*/*R*_free_ | 0.21/0.25 |
| r.m.s. deviations from standard values | |
| bond length (Å) | 0.013 |
| bond angles (º) | 1.30 |
| B-factors (Å^2^) | |
| protein atoms | 44.70 |
| solvent | 47.00 |
| Ramachandran plot | |
| most favoured regions (%) | 99 |
| disallowed regions (%) | 0 |

**Table S3**. Description of yeast strains used in this study.

| strain | description | background | origin |
| --- | --- | --- | --- |
| BY4741 | wild-type | Mat **a**, his3Δ1, leu2Δ0, met15Δ0, ura3Δ0 | ATCC #201 388 |
| AP001 | Lst4-protein A | BY4741, LST4::ProteinA::HIS5 | this study |
| AP002 | Lst7-protein A | BY4741, LST7::ProteinA::HIS5 | this study |
| AP003 | Lst4Δ | BY4741, Lst4::KANMX4 | open biosystems |
| AP004 | Lst7Δ | BY4741, Lst7::KANMX4 | open biosystems |
| AP005 | Lst4ΔLst7Δ | Lst4::KANMX4 Lst7::KANMX4 | this study |
| L40 | yeast two-hybrid | MAT**a** his3Δ200trp1–901 leu2–3112 ade2 LYS2::(4lexAop-HIS3)URA3:: (8lexAop-lacZ)GAL4) | ATCC MYA­3332 |
